# Gut microbiome changes in anti-N-methyl-D-aspartate receptor encephalitis patients

**DOI:** 10.1186/s12883-022-02804-0

**Published:** 2022-07-25

**Authors:** Jingya Wei, Xiao Zhang, Fang Yang, Xiaodan Shi, Xuan Wang, Rong Chen, Fang Du, Ming Shi, Wen Jiang

**Affiliations:** Department of Neurology, Xijing Hospital, Fourth Military Medical University, No. 15 Changle West Street, Xi’an, 710032 Shaanxi province China

**Keywords:** Anti-N-Methyl-D-Aspartate Receptor Encephalitis, Encephalitis, Gastrointestinal Microbiome, Gut-brain axis

## Abstract

**Background:**

Anti-N-methyl-D-aspartate receptor (NMDAR) encephalitis is a type of autoimmune encephalitis. The underlying mechanism(s) remain largely unknown. Recent evidence has indicated that the gut microbiome may be involved in neurological immune diseases via the "gut-brain axis". This study aimed to explore the possible relationship between anti-NMDAR encephalitis and the gut microbiome.

**Methods:**

Fecal specimens were collected from 10 patients with anti-NMDAR encephalitis and 10 healthy volunteers. The microbiome analysis was based on Illumina sequencing of the V3-V4 hypervariable region of the 16S rRNA gene. The alpha, beta, and taxonomic diversity analyses were mainly based on the QIIME2 pipeline.

**Results:**

There were no statistical differences in epidemiology, medication, and clinical characteristics (except for those related to anti-NMDAR encephalitis) between the two groups. ASV analysis showed that *Prevotella* was significantly increased, while *Bacteroides* was reduced in the gut microbiota of the patients, compared with the controls. Alpha diversity results showed a decrease in diversity in the patients compared with the healthy controls, analyzed by the Shannon diversity, Simpson diversity, and Pielou_E uniformity based on the Kruskal–Wallis test (*P* = 0.0342, 0.0040, and 0.0002, respectively). Beta diversity analysis showed that the abundance and composition of the gut microbiota was significantly different between the two groups, analyzed by weighted and unweighted UniFrac distance (*P* = 0.005 and 0.001, respectively).

**Conclusions:**

The abundance and evenness of bacterial distribution were significantly lower and jeopardized in patients with anti-NMDAR encephalitis than in healthy controls. Thus, our findings suggest that gut microbiome composition changes might be associated with the anti-NMDAR encephalitis. It could be a causal agent, or a consequence.

## Background

Anti-N-methyl-D-aspartate receptor (NMDAR) encephalitis is a type of autoimmune encephalitis. Approximately 37% of patients are younger than 18 years of age at the time of presentation [1]. NMDAR encephalitis is a pathological process mediated by anti-NMDAR antibodies that lead to NMDAR dysfunction [2]. However, the causes of abnormal synthesis of anti-NMDAR antibodies remain unclear. It has been reported that 38% of patients are diagnosed with tumor development [3], which implies immune abnormalities. However, the exact relationship between abnormal immune functions and the development of anti-NMDAR antibodies is not fully understood. Previous studies have shown that the gut microbiome is associated with immune abnormalities and autoantibody production [4, 5]. Therefore, we hypothesized that abnormal synthesis of anti-NMDAR antibodies may be related to an imbalance in the gut microbiome.

Gut microbiota are the microorganisms that live in the digestive tracts. It is the most complex and largest microbial ecosystem in the human body and is interdependent with the human body. The gut microbiome is related to the development of multiple sclerosis, Guillain–Barré syndrome, neuromyelitis optica spectrum disorder, and other nervous system diseases [6–8]. An imbalance of the gut microbiome can cause abnormal immune function, leading to the production of specific and non-specific antibodies [4, 9]. Additionally, a group of animal experiments have found that the gut microbiome affects the expression of NMDAR subunits in the amygdale [10], and changes in intestinal microbiota influence the function of NMDAR [11]. These results indicate that an imbalance in the gut microbiome may cause immune function disorders, ultimately leading to anti-NMDAR encephalitis. To understand the relation of the gut microbiome with anti-NMDAR encephalitis, identifying the differences between patients and healthy individuals was necessary. Therefore, this study investigated the changes in gut microbiome of patients with anti-NMDAR encephalitis and healthy controls.

## Methods

### Study subjects

Patients with anti-NMDAR encephalitis in the Department of Neurology of Xijing Hospital between 2017 and 2020 participated in this study. All patients aged between 14 and 60 years were Han people from northwest China. Patients with other types of autoimmune-related encephalitis, such as contactin-associated protein-2, a-amino-3-hydroxy-5-methyl-4-isoxazole receptors, dipeptidyl-peptidase-like protein 6, leucine-rich glioma-inactivated protein-1, lgLON5, gamma-amino butyric acid receptors, or paraneoplastic neurologic syndrome were excluded. Patients with viral, metabolic, or toxic encephalopathy or mental illness were also excluded. Healthy controls were recruited from the health examination center of Xijing Hospital between 2017 and 2020. None of the patients and healthy controls had histories of other autoimmune diseases, and bowel surgery, and neuropsychiatric disorders, with no history of gastroenteritis and probiotic use within the 3 months preceding recruitment. Women who were pregnant or lactating were excluded. Finally, 10 patients were screened. Of these, nine were in the remission stage without relapse and the other had acute encephalitis and was unconscious. Ten healthy controls matched for age, sex, and body mass index (BMI) were enrolled. All patients and controls, or their guardians, signed the informed consent (ethics approval number KY20203218-1). Information on sex, age, height, weight, BMI, medical complications, and medication usage of the patients and controls was collected.

### Specimen collection

Fecal samples from patients and controls that were not in contact with urine or toilet were collected by using sterile swabs. Then these samples were gathered into sterile drying tubes within minutes to hours of deposition, remained untreated and frozen at -80°C immediately in most cases. The samples that could not be stored at -80°C immediately were refrigerated at 4°C until they were transferred to -80°C (typically within 6 h). Diarrheal feces, purulent blood, and mucus feces were not collected. The samples were remained frozen until DNA extraction.

### DNA extraction and 16S rRNA sequencing

Total DNA was extracted from fecal samples using the QIAamp DNA Stool Mini Kit (QIAGEN, Hilden, Germany) according to the manufacturer’s instructions. The V3-V4 regions of the 16S rRNA gene were selected for PCR amplification using 27F/335R, specific primers with barcodes, Phusion High-Fidelity PCR Master Mix with GC Buffer, and high-fidelity PCR amplification enzyme (New England Biolabs, Boston, MA, USA). The TruSeq DNA PCR-Free Sample Preparation Kit (Illumina, San Diego, CA, USA) was used for library construction. The library was quantified using Qubit (Thermo Fisher Scientific, Waltham, MA, USA) and quantitative-PCR. The HiSeq2500 PE250 system (Illumina) was used for high-throughput sequencing. The bacterial amplicon sequences were processed using the QIIME2 (ver. 2019.4) pipeline [12]. Raw data were de-multiplexed, filtered, and denoised, and chimeras were removed. The resulting data were assigned to amplicon sequence variats (ASVs) using the DADA2 (ver. 1.10.0) plugin [13]. Multiple sequence alignments were performed using MAFFT (ver. 7.110) and filtered to remove highly variable positions [14]. FastTree 2 (ver. 2.1.10) was used to construct and root a phylogenetic tree [15]. Taxonomic classification was conducted using a pretrained naïve Bayes classifier trained on Greengenes (ver. 13.8) database for the 16S rRNA region spanning the V3-V4 region. The 16S rRNA data were deposited in OMIX, China National Center for Bioinformation/Beijing Institute of Genomics, Chinese Academy of Sciences (https://ngdc.cncb.ac.cn/omix: accession no. OMIX876).

### Statistical analyses

The microbial composition at each classification level (phylum, class, order, family, and genus) was determined. Alpha and beta diversities are commonly used measures of species diversity and composition in microbiome studies. Alpha diversity quantifies the overall species richness and diversity. Beta diversity gives information on the differences of microbiota composition and abundance [16]. In this study, alpha diversity was assessed using QIIME2 based on the Pielou_E uniformity Shannon diversity, and Simpson diversity indexes. The measured alpha diversity metrics were compared between patient groups. The Wilcoxon/Kruskal–Wallis Rank sum test was used to test the significance. Beta diversity was also calculated using QIIME2 based on weighted and unweighted UniFrac distances. PERMANOVA, and ANOSIM were applied to compare the significance of beta diversity differences between the groups since the two methods have different emphases. ANOSIM was used to assess clustering of samples. PERMANOVA was used to assess the amount of variance of each variable that can explain the distances between the samples. Principal coordinate analysis based on weighted and unweighted UniFrac distances was also performed. To evaluate taxonomic differences between the groups, linear discriminant analysis (LDA) combined with effect size (LEfSe 1.0) was applied. The plots were made by the ggplot2 (ver.3.3.3) R (ver.3.6.1) package.

Clinical data analyses were performed by SPSS 19.0 statistical software (SPSS, Inc., Chicago, IL, USA). Normally distributed data in this study are presented as mean ± SD. The independent sample t-test was used for comparison between the two groups of measurement data with normal distribution, and the Mann–Whitney U test was used for measurement data with skewed distribution. The chi-square test was used to calculate information. The Spearman correlation test was used for correlation analysis. *P* < 0.05.

## Results

### Clinical characteristics between patients and healthy controls

The clinical characteristics of the 10 patients with anti-NMDAR encephalitis and the 10 healthy controls are shown in Table 1. The mean age of the patients and controls was 30.4 and 32.2 years, respectively. Two patients with anti-NMDAR encephalitis received immunosuppressant treatment and one patient received antibiotics in the preceding 6 months. Two patients experienced seizures during the disease course. One patient was seizure-free for 4 months; the drug was discontinued 2 weeks before specimen collection. Another patient had 2–3 seizures per month and had been taking sodium valproate (1000 mg/day) in the last 6 months before specimen collection. None of the patients were on a ketogenic diet. None of the participants had diarrhea or were constipated. All the patients and controls had similar dietary preferences, preferring cooked wheaten food, especially noodles, with moderate consumption of vegetables and meat, and less consumption of rice and fruits.

**Table 1:** Clinical characteristics of the participants

		Anti-NMDAR encephalitis (*n*=10)	Healthy controls (*n*=10)	*P*-value
Epidemiology	Age (years ± SD)	30.4± 12.1	32.2 ± 11.6	*0.738*
	Female/male	4/6	6/4	*0.656*
	Weight (kg ± SD)	66.7 ± 8.3	64.8 ± 10.1	*0.658*
	Height (m ± SD)	1.67 ± 0.07	1.68 ± 0.10	*0.782*
	Body mass index (kg/m^2^ ± SD)	23.7 ± 1.3	22.7±2.0	*0.184*
Medication	Antibiotics (last 6 months)	1	0	*1.000*
	Immunosuppression (last 6 months)	2	0	*0.474*
	Hypertensive	0	0	*-*
	Thyroid Dysfunction	0	0	*-*
	Proton pump inhibitor	1	0	*1.000*
	Diabetes	0	0	*-*
	Anticoagulation	0	0	*-*
Medical condition	Seizure (last 6 months)	2	0	*0.474*
	Anti-epileptic drugs	1	0	*1.000*
	Cognitive impaired	2	0	*0.474*
	Unconscious	1	0	*1.000*
	Pregnant	0	0	*-*
	Diarrhea	0	0	*-*
	Constipation	0	0	*-*

*NMDAR* N-methyl-D-aspartate receptor

### Significant changes of *Prevotella* and *Bacteroides* in patients

**Fig. 1:**
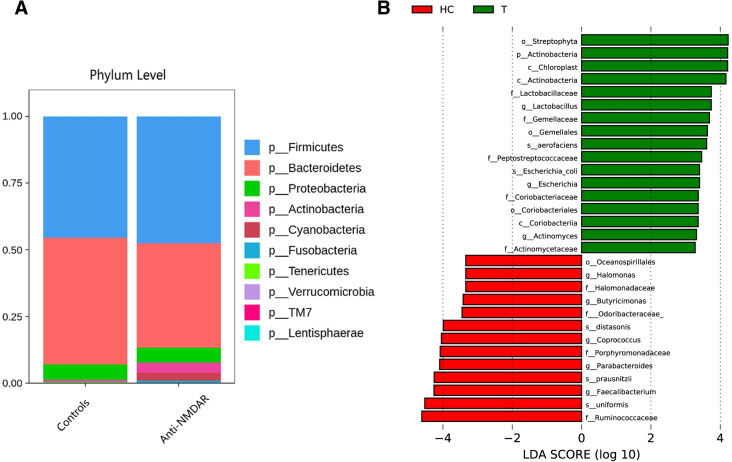
**A** Gut microbiota composition in controls and anti-NMDAR encephalitis patients. **B** Lefse-based comparison of patients and controls

The taxonomic results showed that the genus Prevotella was significantly increased (13.21% vs. 5.36%, *P*=0.048, by Wilcoxon test) and Bacteroides was considerably reduced (27.31% vs. 42.53%, *P*=0.024, by Wilcoxon test) in the patients compared to the controls (shown in Fig. 1A). The collective results demonstrated a significant difference in the bacterial types of gut microbiota between patients with anti-NMDAR encephalitis and healthy controls.

LEFse showed decreased abundances of bacteria (such as Oceanospirillales, *Halomonas*, Halomonadaceae, *Butyricimonas*, Odoribacteraceae, *Coprococcus*, Porphyromonadaceae, *Parabacteroides*, *Faecalibacterium*, and Ruminococcaceae) and increased abundance of bacteria (such as Streptophyta, Lactobacillaceae, *Lactobacillus*, Gemellaceae, Gemellales, Peptostreptococcaceae, Coriobacteriaceae, Coriobacteriales, *Actinomyces*, and Actinomycetaceae) in anti-NMDAR encephalitis patients relative to healthy controls. There were 30 bacterial taxa showing distinct relative abundances between the two groups (LDA score > 2.0, *P*< 0.05, shown in Fig. 1B). LEFse analysis at the genus level had the similar results to those at a mix of different levels (data not shown).

### Reduced alpha diversity and altered microbial composition in anti-NMDAR encephalitis patients

**Fig. 2:**
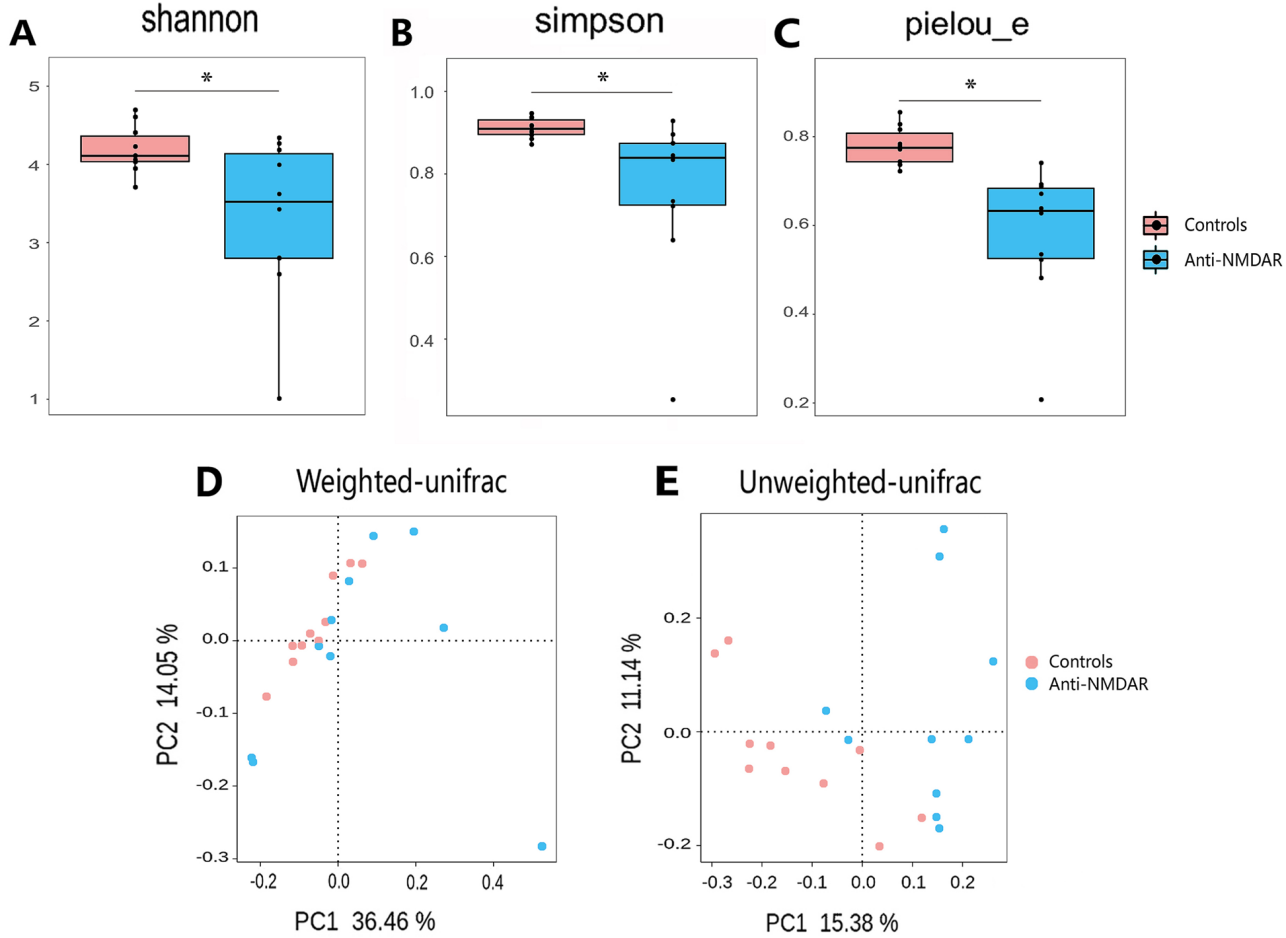
Results of α and β diversity. **A** Shannon, **B** Simpson and **C** Pielou_e indexes were used to calculate the α diversity. **D** Weighted and **E** unweighted UniFrac distance were used to calculate the β diversity

The Shannon diversity, Simpson diversity, and Pielou_E uniformity indices based on the Kruskal–Wallis test revealed a significant decrease in diversity in the patients compared with the controls (shown in Fig. 2A-C). The Shannon diversity and Simpson diversity indices of healthy controls were higher than those of the patient group (*P* = 0.0342 and 0.0040, respectively; shown in Fig. 2A and B). These results showed that the number of identified ASVs in patients with anti-NMDAR encephalitis was significantly lower than that in the healthy controls. In alpha diversity plots of ASV, the Pielou_E index of healthy controls was higher than that of patients. The evenness of microbe distribution in patients with anti-NMDAR encephalitis was significantly lower than that in healthy controls (*P* = 0.0002, shown in Fig. 2C). To examine whether the gut bacterial communities of patients shift significantly, we compared changes in beta diversity. A Permanova comparing bacterial communities of the patients versus the controls across the whole dataset yielded a significant difference. Weighted and unweighted UniFrac distance calculations showed that the microbial community was significantly different between the two groups (*P* = 0.005 and 0.001, respectively; shown in Fig. 2D and E).

## Discussion

This study found that the abundance and evenness of gut microbiota distribution in the patients with anti-NMDAR encephalitis decreased significantly. *Bacteroides* was significantly reduced while *Prevotella* was significantly increased in the patients. *Bacteroides* is a small gram-negative, obligate anaerobic bacillus. It colonizes the intestine, oral cavity, upper respiratory tract, and reproductive tract. These bacteria can reportedly regulate human immune function and are beneficial in prevention of peripheral and central nervous system sterile inflammatory disorders, such as experimental allergic encephalomyelitis and herpes simplex encephalitis [17]. Evidence of many beneficial functions of Bacteroides strains suggests their intervention capabilities in lipopolysaccharide-induced immune response and gut microbiome shift, even having potential as therapeutic probiotics to prevent inflammatory disorders [18, 19]. The polysaccharide A surface component of *Bacteroides fragilis* is mainly responsible for this protective effect [20]. The expression of polysaccharide A can also protect against central nervous system demyelination [21]. Therefore, reduced *Bacteroides* in anti-NMDAR encephalitis may result in a decline in this protective effect, increasing the chance of the disease.

*Prevotella* was formerly classified in the genus *Bacteroides* and was classified as an independent genus in 1990 [22]. It can regulate immune functions and is beneficial to the nervous system. *Prevotella histicola* suppresses central nervous system inflammatory and demyelinating diseases through the modulation of systemic immune responses [23]. It had been proved can suppress multiple sclerosis as effectively as Interferon-β-1b in patients [24]. *Prevotella* in the human gut microbiota has been proved to be negatively correlated with the number of CD20^+^ B lymphocytes in many diseases [25, 26]. CD20^+^ B lymphocytes is positively correlated with the severity of anti-NMDAR encephalitis [27]. Clinically, the rituximab monoclonal antibody targeting CD20^+^ B lymphocytes is effective in the treatment of anti-NMDAR encephalitis. Thus, we hypothesized that increased *Prevotella* levels may decrease the number of CD20^+^ B lymphocytes, which may be a protective factor against anti-NMDAR encephalitis.

A study involving 23 German patients with anti-NMDAR encephalitis found no difference in the gut microbiome compared to healthy controls [28]. Another study in Southwest China showed that the overall species richness of the microbiota was higher in patients than that in controls [29]. The contradictory results of these studies may be attributed to differences in race, lifestyle, and diet. Germans and denizens of Southwest and Northwest China vary markedly in their eating habits, which could appreciably affect the intestinal microecology. Another important reason for the different findings of the two aforementioned studies may be the differences in the disease stages of the patients. The German study enrolled two patients with acute encephalitis, six in the recovery phase, and 15 in the recovered phase. The study in Southwest China enrolled patients in the acute stage with treatment, with relapse, and without relapse in the remission phase. However, in the present study, most patients were in remission without relapse, and only one patient had acute encephalitis and was unconscious. Untreated anti-NMDAR encephalitis patients were reported to exhibit disturbances in their gut microbial composition [30]. Therefore, the gut microbiota may have different manifestations during different periods of anti-NMDAR encephalitis.

The results of alpha and beta diversities confirmed the difference in the gut microbiota between the patients with anti-NMDAR encephalitis and the controls. The change may be affected by anti-NMDAR encephalitis or may be a reason for immune function change. However, it is still unclear whether this change was the cause or the result. It may also be a result of treatment, because all the patients received corresponding treatment before participating in this study. One patient received antibiotic treatment for pneumonia. The patient was unconscious and only received a small amount of liquid food through the nasogastric tube. Thus, a proton pump inhibitor (PPI) was also used. Both antibiotics and PPI have been reported to alter the microbiome [31]. Two patients received mofetil mycophenolate for disease treatment. This drug MMF reduced gut dysbiosis in rats [32], suggesting that it may alter the human gut microbiome. Thus, mofetil mycophenolate may reduce the differences in the gut microbiome between patients and controls. Additionally, two patients experienced seizures during the course of their disease. One was taking sodium valproate. However, there is little evidence regarding the direct interactions between anti-epileptic drugs and the gut microbiome [33].

This study has several limitations. First, only 10 patients and 10 controls were included. Given the small sample size, the findings may not be representative of changes in the overall population. Second, the selection of individuals for analysis was not random, possibly leading to an inevitable bias. Third, the dietary preferences were collected based on information provided by the patients and so the influence of diet on the results cannot be ruled out. In subsequent studies, to obtain a more generalized result, the sample size should be larger and specific questionnaires or tables should be formulated to evaluate eating habits**.**

## Conclusions

Both the abundance and evenness of bacterial distribution in anti-NMDAR encephalitis patients were significantly lower than those in healthy controls. These findings imply that gut microbiome composition changes might be associated with the anti-NMDAR encephalitis. It could be a causal agent, or a consequence.

## Data Availability

The data that support the findings of this study are available from the OMIX, China National Center for Bioinformation, but restrictions apply to the availability of these data, which were used under license for the current study, and so are not publicly available. Data are however available from the authors upon reasonable request and with permission of China National Center for Bioinformation. Wen Jiang should be contacted if someone wants to request the data.

## Abbreviations

NMDAR: N-methyl-D-aspartate receptor
ASV: Amplicon sequence variat
LEfse: Linear discriminant analysis coupled with effect size measurements
BMI: Body mass index
DNA: Deoxyribonucleic acid
RNA: Ribonucleic acid
LDA: Linear discriminant analysis

## Declarations

Study approval statement: This study protocol was reviewed and approved by the Medical Ethics Committee of the First Affiliated Hospital of the Air Force Medical University, approval number [KY20182024-F-1].

Informed consent was obtained from participants or their legal guardian.

## References

[1] Dalmau J, Armangue T, Planaguma J, Radosevic M, Mannara F, Leypoldt F (2019). An update on anti-NMDA receptor encephalitis for neurologists and psychiatrists: mechanisms and models. Lancet Neurol.

[2] Ladepeche L, Planaguma J, Thakur S, Suarez I, Hara M, Borbely JS (2018). NMDA Receptor Autoantibodies in Autoimmune Encephalitis Cause a Subunit-Specific Nanoscale Redistribution of NMDA Receptors. Cell Rep.

[3] Titulaer MJ, McCracken L, Gabilondo I, Armangue T, Glaser C, Iizuka T (2013). Treatment and prognostic factors for long-term outcome in patients with anti-NMDA receptor encephalitis: an observational cohort study. Lancet Neurol.

[4] Petta I, Fraussen J, Somers V, Kleinewietfeld M (2018). Interrelation of Diet, Gut Microbiome, and Autoantibody Production. Front Immunol.

[5] Kim M, Kim CH (2017). Regulation of humoral immunity by gut microbial products. Gut Microbes.

[6] Probstel AK, Baranzini SE (2018). The Role of the Gut Microbiome in Multiple Sclerosis Risk and Progression: Towards Characterization of the "MS Microbiome". Neurotherapeutics.

[7] Rodriguez Y, Rojas M, Pacheco Y, Acosta-Ampudia Y, Ramirez-Santana C, Monsalve DM (2018). Guillain-Barre syndrome, transverse myelitis and infectious diseases. Cell Mol Immunol.

[8] Cree BA, Spencer CM, Varrin-Doyer M, Baranzini SE, Zamvil SS (2016). Gut microbiome analysis in neuromyelitis optica reveals overabundance of Clostridium perfringens. Ann Neurol.

[9] Kim M, Qie Y, Park J, Kim CH (2016). Gut Microbial Metabolites Fuel Host Antibody Responses. Cell Host Microbe.

[10] Neufeld KM, Kang N, Bienenstock J, Foster JA (2011). Reduced anxiety-like behavior and central neurochemical change in germ-free mice. Neurogastroenterol Motil.

[11] Gronier B, Savignac HM, Di Miceli M, Idriss SM, Tzortzis G, Anthony D (2018). Increased cortical neuronal responses to NMDA and improved attentional set-shifting performance in rats following prebiotic (B-GOS((R))) ingestion. Eur Neuropsychopharmacol.

[12] Bolyen E, Rideout JR, Dillon MR, Bokulich NA, Abnet CC, Al-Ghalith GA (2019). Reproducible, interactive, scalable and extensible microbiome data science using QIIME 2. Nat Biotechnol.

[13] Callahan BJ, McMurdie PJ, Rosen MJ, Han AW, Johnson AJ, Holmes SP (2016). DADA2: High-resolution sample inference from Illumina amplicon data. Nat Methods.

[14] Nakamura T, Yamada KD, Tomii K, Katoh K (2018). Parallelization of MAFFT for large-scale multiple sequence alignments. Bioinformatics.

[15] Price MN, Dehal PS, Arkin AP (2010). FastTree 2--approximately maximum-likelihood trees for large alignments. PLoS One.

[16] Koponen KK, Salosensaari A, Ruuskanen MO, Havulinna AS, Mannisto S, Jousilahti P (2021). Associations of healthy food choices with gut microbiota profiles. Am J Clin Nutr.

[17] Ramakrishna C, Kujawski M, Chu H, Li L, Mazmanian SK, Cantin EM (2019). Bacteroides fragilis polysaccharide A induces IL-10 secreting B and T cells that prevent viral encephalitis. Nat Commun.

[18] Tan H, Zhao J, Zhang H, Zhai Q, Chen W (2019). Novel strains of Bacteroides fragilis and Bacteroides ovatus alleviate the LPS-induced inflammation in mice. Appl Microbiol Biotechnol.

[19] Qu D, Sun F, Feng S, Yu L, Tian F, Zhang H (2022). Protective effects of Bacteroides fragilis against lipopolysaccharide-induced systemic inflammation and their potential functional genes. Food Funct.

[20] Gorreja F, Rush ST, Kasper DL, Meng D, Walker WA (2019). The developmentally regulated fetal enterocyte gene, ZP4, mediates anti-inflammation by the symbiotic bacterial surface factor polysaccharide A on Bacteroides fragilis. Am J Physiol Gastrointest Liver Physiol.

[21] Ochoa-Reparaz J, Mielcarz DW, Ditrio LE, Burroughs AR, Begum-Haque S, Dasgupta S (2010). Central nervous system demyelinating disease protection by the human commensal Bacteroides fragilis depends on polysaccharide A expression. J Immunol.

[22] Shah HN, Collins DM (1990). Prevotella, a new genus to include Bacteroides melaninogenicus and related species formerly classified in the genus Bacteroides. Int J Syst Bacteriol.

[23] Mangalam A, Shahi SK, Luckey D, Karau M, Marietta E, Luo N (2017). Human Gut-Derived Commensal Bacteria Suppress CNS Inflammatory and Demyelinating Disease. Cell Rep.

[24] Shahi SK, Jensen SN, Murra AC, Tang N, Guo H, Gibson-Corley KN (2020). Human Commensal Prevotella histicola Ameliorates Disease as Effectively as Interferon-Beta in the Experimental Autoimmune Encephalomyelitis. Front Immunol.

[25] Shahi SK, Freedman SN, Murra AC, Zarei K, Sompallae R, Gibson-Corley KN (2019). Prevotella histicola, A Human Gut Commensal, Is as Potent as COPAXONE(R) in an Animal Model of Multiple Sclerosis. Front Immunol.

[26] Schwenger KJP, Chen L, Chelliah A, Da Silva HE, Teterina A, Comelli EM (2018). Markers of activated inflammatory cells are associated with disease severity and intestinal microbiota in adults with nonalcoholic fatty liver disease. Int J Mol Med.

[27] Forsthuber TG, Cimbora DM, Ratchford JN, Katz E, Stuve O (2018). B cell-based therapies in CNS autoimmunity: differentiating CD19 and CD20 as therapeutic targets. Ther Adv Neurol Disord.

[28] Herken J, Bang C, Ruhlemann MC, Finke C, Klag J, Franke A, et al. Normal gut microbiome in NMDA receptor encephalitis. Neurol Neuroimmunol Neuroinflamm. 2019;6(6):e632. 10.1212/NXI.0000000000000632 PMC6857909 31624178

[29] Gong X, Liu X, Li C, Chen C, Lin J, Li A (2019). Alterations in the human gut microbiome in anti-N-methyl-D-aspartate receptor encephalitis. Ann Clin Transl Neurol.

[30] Chen H, Chen Z, Shen L, Wu X, Ma X, Lin D (2020). Fecal microbiota transplantation from patients with autoimmune encephalitis modulates Th17 response and relevant behaviors in mice. Cell Death Discov.

[31] Reveles KR, Ryan CN, Chan L, Cosimi RA, Haynes WL (2018). Proton pump inhibitor use associated with changes in gut microbiota composition. Gut.

[32] Robles-Vera I, de la Visitacion N, Toral M, Sanchez M, Gomez-Guzman M, Jimenez R (2021). Mycophenolate mediated remodeling of gut microbiota and improvement of gut-brain axis in spontaneously hypertensive rats. Biomed Pharmacother.

[33] Dahlin M, Prast-Nielsen S (2019). The gut microbiome and epilepsy. EBioMedicine.

